# A Note with a Zoological Interest

**Published:** 1919-12-20

**Authors:** 


### A NOTE WITH A ZOOLOGICAL INTEREST.

To wander into the realms of comparative science, and there to study a few of the many complex problems intimately associated therein, forms a most fascinating and instructive recreation. We all know the sounds of unrestrained laughter that follow the antics of the monkeys at the Zoological Gardens, or the thrills of shuddering horror that pervade one's whole being when watching the slow, stealthy, silent movements of the tiger. Yet there are probably comparatively few people who will take the trouble to notice, with an observant eye and reasoning mind, the sly, cunning motions of their own cats at home, whose movements, though generally harmless, are yet similar in origin to those of the fierce inhabitants caged in Regent's Park; or to consider whether what, to them, seems buffoonery is not, perhaps, common sense to the lower animal.

### The Third Eyelid.

A few weeks ago, when delivering the opening address at the Middlesex Hospital, London, Sir John Bland Sutton, Senior Surgeon to that Institution, took his hearers, in his own inimitable way, into a land of anatomical interest and promise. He allocated some of his remarks to a discussion on the so-called "Third Eyelid," illustrating his statements by means of large coloured diagrams. The short epitome below may perhaps help to throw a little light on to the meaning and uses of this "Third Eyelid."

The following muscles are found in many animals, but not usually in man; I., retractor bulbi, or choanoid muscle; this is a funnel-shaped sheet of muscle lying within the recti muscles (viz., those muscles which move the eyeball), and it is inserted into the posterior moiety of the eye. It is sometimes present in man, but when not, as is generally the case, it is represented, according to Sir John Bland Sutton, by a constant structure called Tenon's capsule, which serves the purpose of supporting the eyeball. Now it has been stated that the function of the choanoid muscle in animals is to prevent the eye from falling out; this latter statement, however, is a very difficult one to believe, for several reasons, which space will not allow for discussion in this paper.

2. Nictitating muscles; these are divided into parts, viz., (*a*) M. bursalis, (*b*) M. pyramidalis oculi. Both (*a*) and (*b*) may occur separately or together; they are found in most lizards, in turtles, tortoises, crocodiles, alligators, and birds; and present different arrangements in different species.

In lizards the M. bursalis is short and thick and arises from the inner and posterior wall of the orbit. Again, a tendon is attached to the inner wall of the orbit in front, and passes backwards through the sheath formed by the bursalis; that is to say, that the M. bursalis ends in a sheath, and thus, after passing through this, the tendon is inserted into the margin of the nictitating membrane.

In lizards, etc., the M. pyramidalis rises from the inner side of the eyeball, then curves over the optic nerve and the eyeball to be inserted into the nictitating membrane; and also into the lower lid.

In crocodiles there is an almost similar arrangement, viz., the M. pyramidalis is inserted into the nictitating membrane, but it has no attachment to the lower lid. The M. buralis exists in birds, and arises from the upper part of the sclerotic and, passing backwards, ends in a sheath which embraces the tendon of M. pyramidalis; whilst the M. pyramidalis itself arises from the inner and under aspect of the sclerotic (the outer coat of the eyeball), and ends in a tendon which passes over the upper and outer part of the sclerotic to become attached to the nictitating membrane. Thus, the resultant force, on muscular contraction, pulls the nictitating membrane upwards, outwards, and backwards across the front of the eye.

### The Provisions of Nature.

Animals travelling under water or living in sandy districts, or burrowing in sand, possess a transparent nictitating membrane. It has been found that these classes of the animal world are frequently devoid of the muscles which elevate, or depress, the eyelids. The clearing of the conjunctiva (a thin transparent covering over the front of the eye, and also lining the inner surfaces of the lids), carried out in man, and many animals, by the opening and closing of the lids, is then performed by the nictitating membrane.

It is most interesting to note that all the special muscles mentioned above are supplied by the same cranial nerve—viz., the sixth—on the same side of the body, and that therefore they all originate from the same segment of the body. The nictitating membrane is well seen in the horse, turkey, parrot, pigeon, lizard, etc. One authority has suggested that when the horse draws this membrane across his eye he is giving expression to his feelings of affection for the opposite equine sex, but this parallel it is a little difficult seriously to believe.

Nevertheless, these excursions into comparative anatomy are more than interesting, and it is to be regretted that they do not more frequently appear in the medical Press. Not only interest, but true worth may they hold, and as in many other regions, so in this do we owe a great debt of gratitude to that supreme genius, Huxley, whose researches have proved to be of such inestimable value to the world.

